# Predicting lethal courses in critically ill COVID-19 patients using a machine learning model trained on patients with non-COVID-19 viral pneumonia

**DOI:** 10.1038/s41598-021-92475-7

**Published:** 2021-06-24

**Authors:** Gregor Lichtner, Felix Balzer, Stefan Haufe, Niklas Giesa, Fridtjof Schiefenhövel, Malte Schmieding, Carlo Jurth, Wolfgang Kopp, Altuna Akalin, Stefan J. Schaller, Steffen Weber-Carstens, Claudia Spies, Falk von Dincklage

**Affiliations:** 1 Charité – Universitätsmedizin Berlin, corporate member of Freie Universität Berlin, Humboldt-Universität zu Berlin, and Berlin Institute of Health, Department of Anesthesiology and Operative Intensive Care Medicine (CCM, CVK), Charitéplatz 1, 10117 Berlin, Germany; 2 Charité – Universitätsmedizin Berlin, corporate member of Freie Universität Berlin, Humboldt-Universität zu Berlin, and Berlin Institute of Health, Institute of Medical Informatics, Berlin, Germany; 3 Einstein Center Digital Future, Berlin, Germany; 4 Charité – Universitätsmedizin Berlin, corporate member of Freie Universität Berlin, Humboldt-Universität zu Berlin, and Berlin Institute of Health, Klinik für Neurologie mit Experimenteller Neurologie, Berlin, Germany; 5 Max‐Delbrück‐Center for Molecular Medicine in the Helmholtz Association (MDC), Berlin Institute for Medical Systems Biology (BIMSB), Berlin, Germany; 6 Physikalisch-Technische Bundesanstalt Braunschweig und Berlin, Department of Mathematical Modelling and Data Analysis, Berlin, Germany; 7 Technische Universität Berlin, Uncertainty, Inverse Modeling and Machine Learning Group, Berlin, Germany

**Keywords:** Infectious diseases, Prognosis, Machine learning, Predictive medicine

## Abstract

In a pandemic with a novel disease, disease-specific prognosis models are available only with a delay. To bridge the critical early phase, models built for similar diseases might be applied. To test the accuracy of such a knowledge transfer, we investigated how precise lethal courses in critically ill COVID-19 patients can be predicted by a model trained on critically ill non-COVID-19 viral pneumonia patients. We trained gradient boosted decision tree models on 718 (245 deceased) non-COVID-19 viral pneumonia patients to predict individual ICU mortality and applied it to 1054 (369 deceased) COVID-19 patients. Our model showed a significantly better predictive performance (AUROC 0.86 [95% CI 0.86–0.87]) than the clinical scores APACHE2 (0.63 [95% CI 0.61–0.65]), SAPS2 (0.72 [95% CI 0.71–0.74]) and SOFA (0.76 [95% CI 0.75–0.77]), the COVID-19-specific mortality prediction models of Zhou (0.76 [95% CI 0.73–0.78]) and Wang (laboratory: 0.62 [95% CI 0.59–0.65]; clinical: 0.56 [95% CI 0.55–0.58]) and the 4C COVID-19 Mortality score (0.71 [95% CI 0.70–0.72]). We conclude that lethal courses in critically ill COVID-19 patients can be predicted by a machine learning model trained on non-COVID-19 patients. Our results suggest that in a pandemic with a novel disease, prognosis models built for similar diseases can be applied, even when the diseases differ in time courses and in rates of critical and lethal courses.

## Introduction

The coronavirus disease 2019 (COVID-19) pandemic poses a major threat to global health. Despite all efforts to slow the spreading and contain the disease, healthcare systems in countries all over the world have been overwhelmed with high demands for critical care resources. To manage these demands in the best possible way and to enable an effective and efficient allocation of critical care resources, prognosis models for individual disease courses and outcomes are essential. Accordingly, several prognosis models for critical and lethal courses in critically ill COVID-19 patients have been published over the course of the year^1–8^.

The reported predictors for lethal courses in COVID-19 patients can be divided into seven groups, including (1) demographic features like age and gender, (2) comorbidities like COPD, obesity, hypertension and diabetes, (3) radiological signs of disease severity like multi-lobular infiltration, (4) blood infection markers and infection associated blood count parameters like C-reactive protein, procalcitonin and lymphocyte counts, (5) other laboratory blood markers associated with organ distress like lactate dehydrogenase, bilirubin or blood urea nitrogen, (6) direct clinical signs of organ failure like respiratory rate, blood oxygenation or blood pressure and (7) intensive care treatment measures as indirect markers of organ failure like catecholamine doses or ventilation parameters.

Interestingly, the predictors that were identified to indicate critical and lethal courses in COVID-19 patients are very similar to those applied in models for the prediction of lethal courses in critically ill non-COVID-19 viral pneumonia patients^9–13^. This similarity is not entirely surprising, as the fundamental pathophysiological mechanisms of organ failure in those patients developing a critical or lethal course appear relatively similar between COVID-19 and other types of viral pneumonia, even though the rate of patients developing a critical or lethal course and the time frame of such courses may differ profoundly.

Such pathophysiological similarities of critical and lethal courses between intensive care patients with different types of viral pneumonia might allow to transfer knowledge obtained on one type of viral pneumonia to other types, even though they differ in mortality rates and time courses. Especially in a pandemic situation with a new type of disease, such knowledge transfer might be highly beneficial, as it would bridge the critical early phase by allowing the use of prediction models built for similar diseases until first models based on data of the actual disease are available.

To test our hypothesis that models developed to predict lethal courses for one type of viral pneumonia also allow to predict lethal courses for another type of viral pneumonia, even when the specific diseases differ in lethality rate and time courses, we performed this study. To specifically address the pandemic scenario, we investigated how well lethal courses in critically ill COVID-19 patients can be predicted by a machine learning model trained on data of critically ill patients with non-COVID-19 viral pneumonia.

## Results

### Patient sample

**Figure 1:**
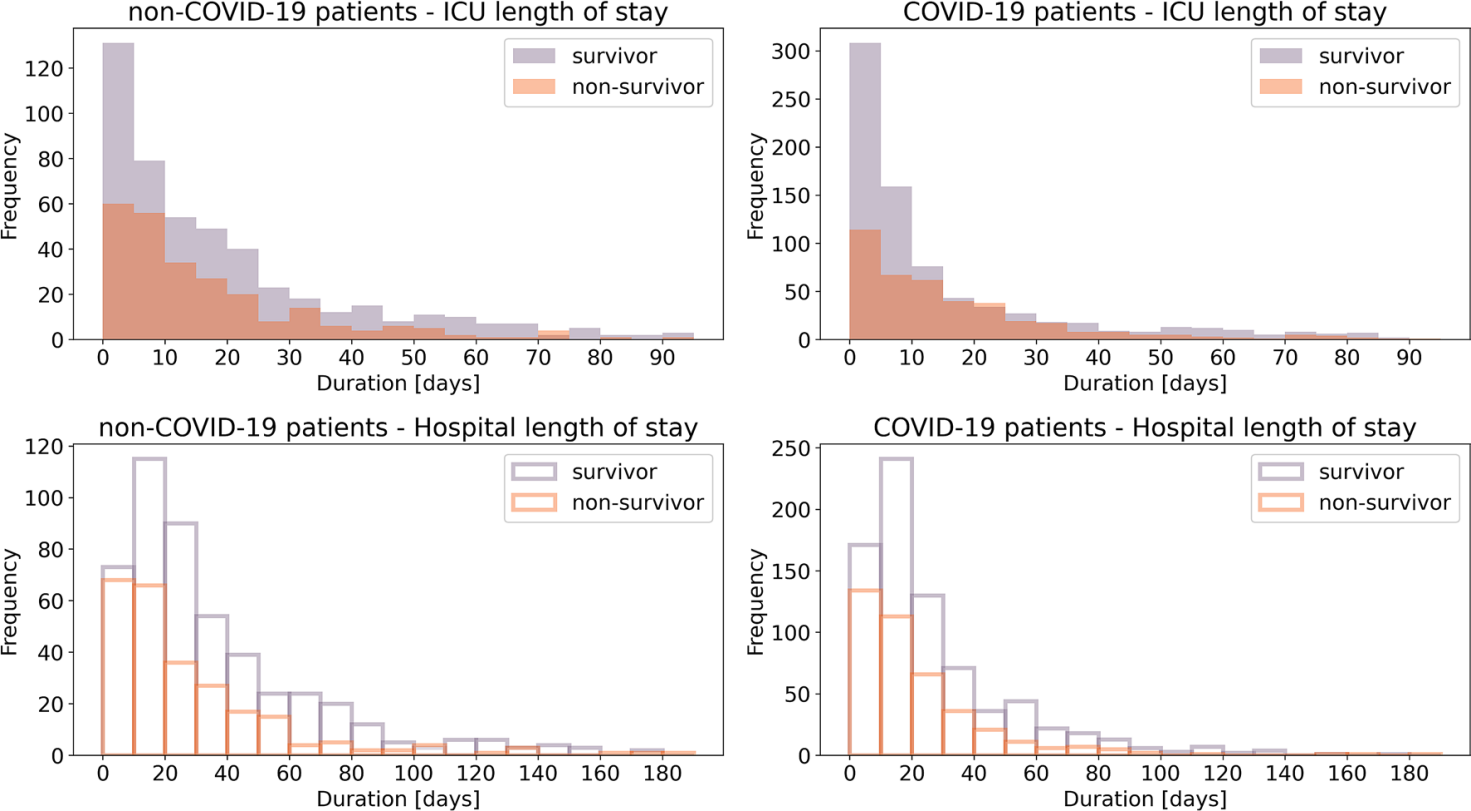
**Durations of ICU treatment and hospitalization of all formerly treated patients.** Shown are the histograms of length of stay in intensive care units (top) and total length of stay in the hospital (bottom) for critically ill non-COVID-19 patients (left) and critically ill COVID-19 patients (right), separately for survivors (purple) and non-survivors (orange). 7 (1) non-COVID-19 (COVID-19) patients with more than 200 days in the hospital and 20 (11) non-COVID-19 (COVID-19) patients with more than 100 days in an ICU are not shown in this illustration as they are out of the depicted axis range.

Of the 749 critically ill non-COVID-19 viral pneumonia patients for which we extracted data, 31 patients were excluded as their ICU treatment was shorter than 24 h or as they were also tested positive for SARS-CoV-19, leaving 718 patients (473 survivor/245 non-survivor) with a median ICU length of stay of 13 d (IQR 5–28 d) for a total of 16,180 time bins of 24 h duration for model training (Fig. 1, Table 1).

**Table 1:** **Patient characteristics.**

	Non-COVID-19 patients (training dataset)	COVID-19 patients (test dataset)
n	718	1054
Deceased	245 (34%)	369 (35%)
Age [a]	62.0 (50.0–73.0)	67.0 (57.0–77.0)
Sex	282 female (39%)	333 female (32%)
BMI [kg/m^2^]	25.7 (22.3–29.6)	27.8 (24.7–32.7)
Asthma	18 (3%)	51 (5%)
Carcinoma	171 (24%)	67 (6%)
Cardiovascular diseases	370 (52%)	444 (42%)
COPD	204 (28%)	142 (13%)
Coronary heart disease	152 (21%)	217 (21%)
Diabetes	340 (47%)	462 (44%)
Hypertension	402 (56%)	690 (65%)
Chronic kidney diseases	179 (25%)	194 (18%)
Lung diseases	267 (37%)	229 (22%)
Malnutrition	201 (28%)	182 (17%)
Metabolic disorders	477 (66%)	608 (58%)
Obesity	85 (12%)	129 (12%)
Pulmonary fibrosis	59 (8%)	54 (5%)
Pulmonary hypertension	320 (45%)	340 (32%)
Stroke	85 (12%)	142 (13%)

The table shows descriptive statistics of the non-COVID-19 patient training dataset and the COVID-19 patients test dataset (median (IQR) for continuous variables; n cases (percentage of group total) for binary variables).

For the COVID-19 dataset, we extracted the data of 1176 critically ill patients with completed cases. Of these, 122 were excluded as their ICU treatment was shorter than 24 h or as they were also tested positive for another virus possibly causing pneumonia, leaving 1054 patients (685 survivor/369 non-survivor) with a median ICU length of stay of 9 d (IQR 4–22 d) for a total of 18,521 time bins of 24 h duration for model testing (Fig. 1, Table 1).

### Prediction model performance

**Figure 2:**
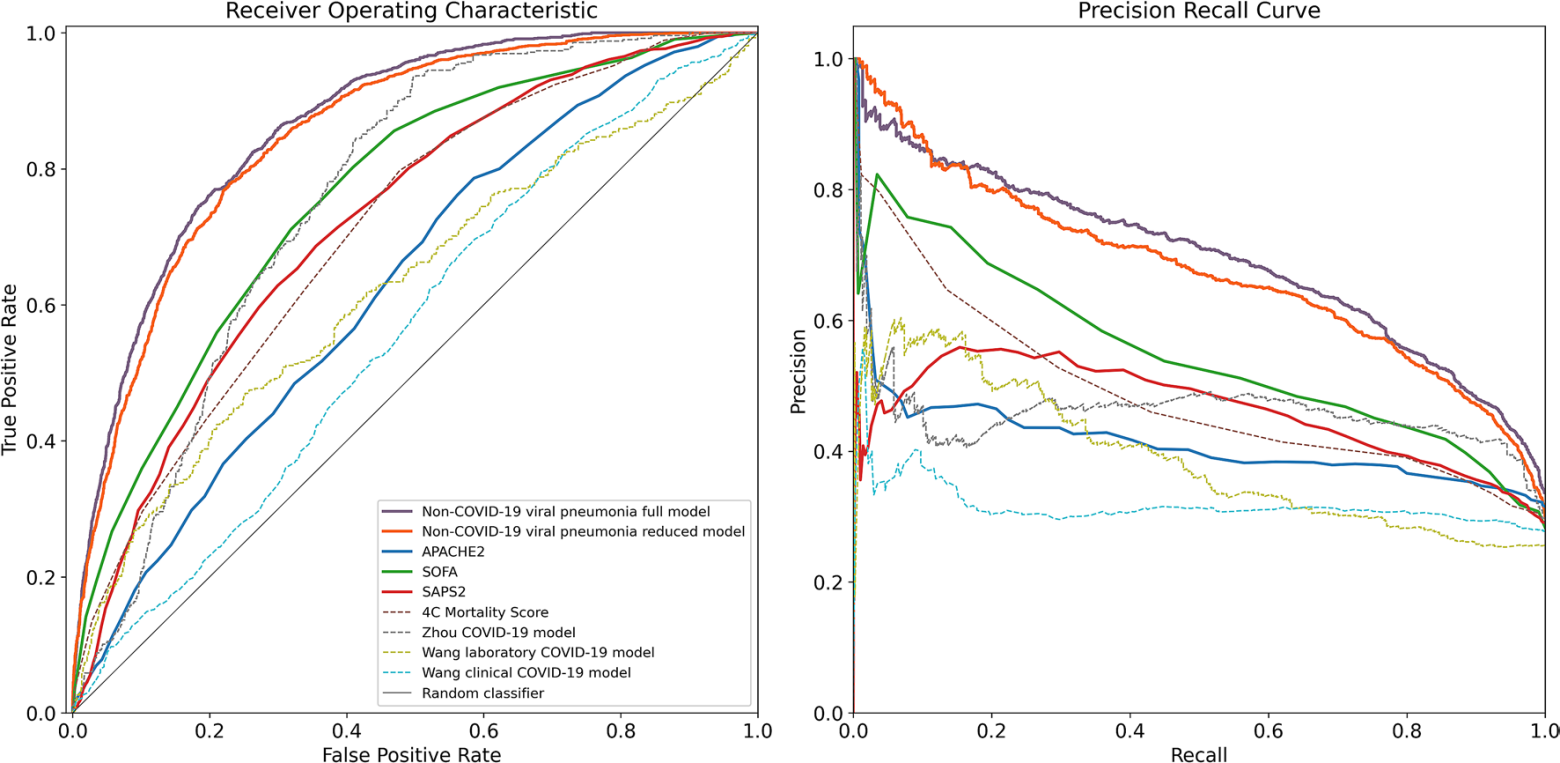
**Performance metrics of the non-COVID-19 viral pneumonia mortality prediction models, clinical scores and previously published COVID-19 mortality prediction models.** Shown are the receiver operating characteristics (left) and precision-recall (right) curves for the full (purple) and reduced (orange) non-COVID-19 viral pneumonia mortality prediction model and for the clinical scores APACHE2 (blue), SOFA (green), SAPS2 (red) for the prediction of mortality within the next 5 days in COVID-19 patients across all 24 h time bins of each patient’s stay on the ICU, weighted inversely by the number of time bins per patient. Additionally shown are the ROC and PRC curves of previously published COVID-19 mortality prediction models (dashed lines) and the performance of a random classifier (solid gray).

The multivariate non-COVID-19 viral pneumonia gradient boosted tree model using the full feature set as well as the reduced model that only included the 20 features with the highest importance on the training dataset both showed a significantly better predictive performance than any of the clinical scores APACHE2, SAPS2 and SOFA, and the previously published prediction models (Fig. 2, Table 2).

**Table 2:** **Performance metrics.**

	Non-COVID-19 viral pneumonia full model	Non-COVID-19 viral pneumonia reduced model	APACHE2	SOFA	SAPS2	4C Mortality Score	Zhou COVID-19 model	Wang laboratory COVID-19 model	Wang clinical COVID-19 model
auROC	0.86 (0.86–0.87)	0.85 (0.84–0.86)	0.63 (0.61–0.65)	0.76 (0.75–0.77)	0.72 (0.71–0.74)	0.71 (0.70–0.72)	0.76 (0.73–0.78)	0.62 (0.59–0.65)	0.56 (0.55–0.58)
auPRC	0.69 (0.67–0.71)	0.68 (0.65–0.70)	0.41 (0.39–0.44)	0.53 (0.51–0.56)	0.46 (0.44–0.48)	0.46 (0.43–0.48)	0.46 (0.42–0.50)	0.39 (0.35–0.43)	0.32 (0.30–0.34)
F1 score	0.67 (0.66–0.68)	0.66 (0.64–0.67)	0.51 (0.49–0.52)	0.56 (0.54–0.58)	0.53 (0.51–0.54)	0.50 (0.48–0.51)	0.58 (0.55–0.61)	0.43 (0.41–0.47)	0.44 (0.43–0.46)
PPV/Precision	0.61 (0.59–0.63)	0.57 (0.55–0.62)	0.38 (0.35–0.39)	0.45 (0.43–0.50)	0.44 (0.39–0.47)	0.41 (0.40–0.43)	0.42 (0.40–0.46)	0.33 (0.28–0.43)	0.29 (0.28–0.32)
NPV	0.89 (0.88–0.90)	0.90 (0.88–0.91)	0.80 (0.79–0.84)	0.87 (0.83–0.88)	0.83 (0.82–0.86)	0.82 (0.81–0.83)	0.95 (0.90–0.96)	0.81 (0.79–0.84)	0.83 (0.80–0.85)
Sensitivity	0.74 (0.72–0.77)	0.77 (0.70–0.79)	0.76 (0.74–0.88)	0.75 (0.63–0.80)	0.65 (0.60–0.77)	0.62 (0.60–0.64)	0.93 (0.83–0.95)	0.62 (0.46–0.81)	0.92 (0.80–0.93)
Specificity	0.82 (0.80–0.84)	0.78 (0.76–0.84)	0.44 (0.28–0.47)	0.64 (0.59–0.74)	0.67 (0.53–0.73)	0.66 (0.65–0.67)	0.50 (0.49–0.61)	0.57 (0.31–0.78)	0.15 (0.14–0.32)
Threshold@max F1	0.15 (0.13–0.16)	0.16 (0.15–0.21)	20.00 (16.00–21.00)	7.00 (6.00–9.00)	43.00 (39.00–45.00)	13.00 (13.00–13.00)	21.75 (21.51–25.43)	− 15.82 (− 19.50–− 13.12)	5.53 (5.53–6.57)
n time bins	18,521	18,521	13,361	17,255	17,245	18,521	4774	4480	18,521
n patients	1054	1054	607	921	925	1054	278	253	1054
Brier score	0.15 (0.15–0.16)	0.15 (0.15–0.16)							

The table shows the area under the ROC (auROC) and the area under the precision-recall curve (auPRC) as threshold-independent performance metrics and the F1 score, positive predictive value (PPV)/precision, negative predictive value (NPV), sensitivity/recall and specificity at a classifier threshold that maximizes the F1 score (Threshold@max F1) for each of the models/scores applied to the COVID-19 viral pneumonia patients test dataset for the prediction of mortality within the next 5 days across all 24 h time bins of each patient’s stay on the ICU, weighted inversely by the number of time bins per patient. Additionally shown are the number of included time bins (note that there are usually multiple time bins per patient) and the number of included unique patients for each of the models and the Brier score for the two models that output a probability score for the prediction.

**Figure 3:**
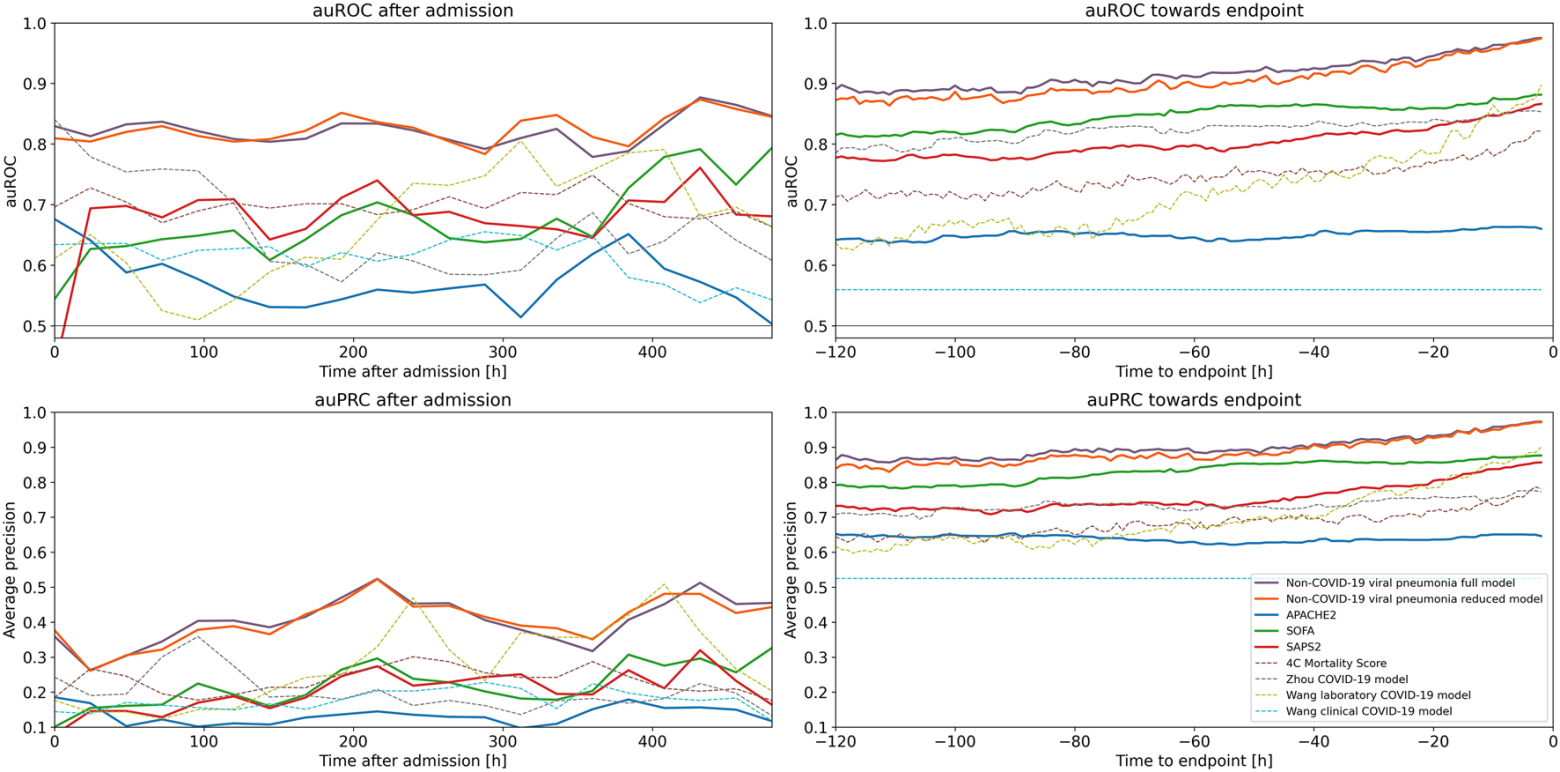
**Time courses of the area under the ROC curves (auROC) and area under the precision recall curve (auPRC) of the non-COVID-19 viral pneumonia mortality prediction model, clinical scores and previously published COVID-19 mortality prediction models.** Shown are the auROC (top) and auPRC (bottom) time courses between admission and 20 days after admission (left) and between 120 and 1 h before the endpoint (death/control endpoint; right) for the full (purple) and reduced (orange) non-COVID-19 viral pneumonia mortality prediction models and for the clinical scores APACHE2 (blue), SOFA (green), SAPS2 (red) for the prediction of mortality within the next 5 days in COVID-19 patients. Prediction windows for the time courses after admission were 24 h and prediction windows for the time courses before the endpoints were 1 h. Additionally shown are the ROC and PRC curves of previously published COVID-19 mortality prediction models (dashed lines) and the performance of a random classifier (solid gray).

The time courses of prediction metrics for all models that used time-varying variables increased with increasing time after admission, and reached their maximum towards the endpoint (Fig. 3). Throughout the first day after admission to the end of stay, both the full and the reduced model outperformed all clinical scores and previously published COVID-19 prediction models. Additionally, the performance of the reduced model did not systematically differ from that of the full model during the first days after admission. However, it was reduced 5 days before the endpoint, but approximated the performance of the full model towards the endpoint.

### Clinical features of the reduced model

**Figure 4:**
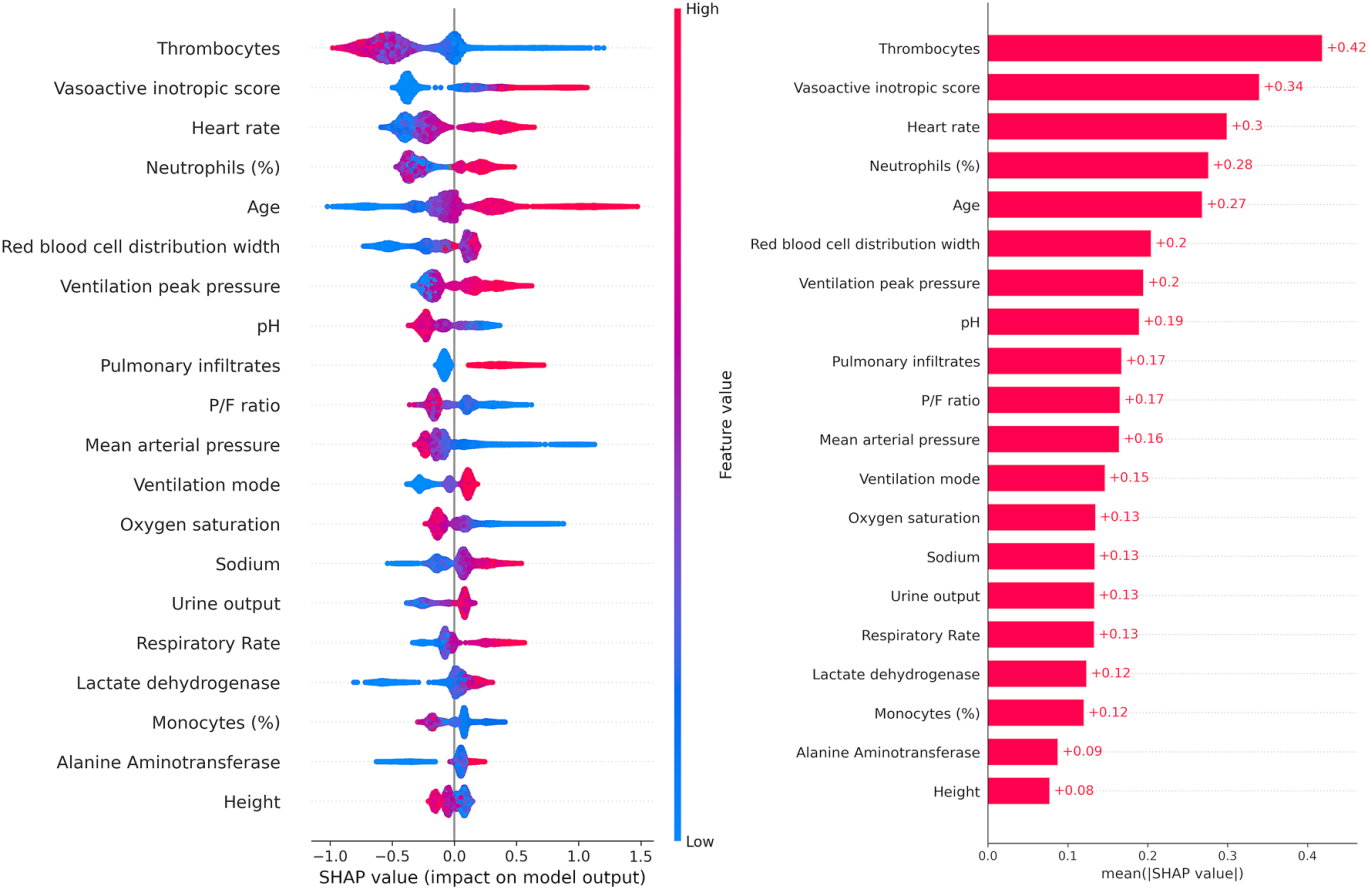
**Impact of clinical features on the prediction of mortality in COVID-19 patients (SHAP values).** Left: Shown is the impact of feature values on the reduced models’ output for prediction of mortality of COVID-19 patients within the next 5 days. Each point represents a patient’s feature value (color-coded from blue for a low feature value to red for high feature values). Negative impact (left to the vertical line) of a feature value represents an impact towards the prediction of survival, positive impact (right to the vertical line) represents an impact towards prediction of non-survival. For example, high thrombocytes concentration levels (red) are associated with a higher probability of survival whereas low mean arterial blood pressure (blue) is associated with a lower probability of survival. Right: Mean absolute impact of each clinical feature on the model output.

From the 251 features of the full model, we determined those 20 unique clinical features that showed the highest feature importance as quantified by the mean absolute SHAP values on the non-COVID-19 viral training dataset (Fig. 4). Most of these features showed a significant difference between patients who deceased within the next 5 days and patients who survived the next 5 days already within the first 24 h after admission, both for the non-COVID-19 patients training and the COVID-19 patients test dataset (Table 3).

**Table 3:** **Univariate analyses of the clinical features for the reduced multivariate viral pneumonia prediction model for data from the first 24 h after admission.**

Feature	Percentile^#^	Non-COVID-19 intensive care patients (training dataset)					COVID-19 intensive care patients (test dataset)				
		Survivors		Deceased		p value	Survivors		Deceased		p value
		n	Median (IQR)/count (%)	n	Median (IQR)/count (%)		n	Median (IQR)/count (%)	n	Median (IQR)/count (%)	
Age [a]	50th	656	62.0 (48.0–72.0)	62	66.5 (56.5–75.0)	0.0043	961	66.0 (56.0–76.0)	93	73.0 (65.0–82.0)	< 0.0001
Vasoactive inotropic score	50th	656	4.0 (0.0–17.9)	62	25.0 (6.0–56.0)	< 0.0001	961	0.0 (0.0–5.0)	93	4.1 (0.0–25.0)	< 0.0001
Thrombocytes [/nl]	10th	654	163.5 (94.2–245.8)	62	80.5 (29.5–160.8)	< 0.0001	955	213.0 (158.5–286.5)	93	178.0 (104.0–238.0)	< 0.0001
Heart rate [1/min]	10th	650	81.8 (69.3–95.1)	61	89.1 (74.1–100.1)	0.031	959	70.0 (61.0–81.0)	93	78.0 (64.0–85.0)	0.0009
Neutrophils (%) [%]	10th	309	80.4 (69.2–87.4)	27	84.9 (79.1–92.9)	0.0263	731	80.9 (73.5–86.3)	55	84.0 (77.2–88.8)	0.0066
Urine output [ml/d]	50th	656	− 1160.0 (− 2032.5–− 489.0)	62	− 845.0 (− 1750.0–− 238.8)	0.0519	961	− 1200.0 (− 1860.0–− 700.0)	93	− 750.0 (− 1320.0–− 200.0)	< 0.0001
Mean arterial pressure [mmHg]	10th	650	66.8 (61.0–72.4)	61	62.0 (56.5–65.9)	< 0.0001	959	68.0 (62.0–75.0)	93	62.0 (57.0–66.0)	< 0.0001
P/F ratio [mmHg]	10th	557	149.1 (106.1–199.2)	58	112.1 (79.1–150.0)	< 0.0001	780	130.1 (98.8–173.1)	76	106.6 (82.9–145.3)	0.0001
Respiratory Rate [1/min]	90th	637	22.5 (18.4–27.6)	60	24.0 (20.0–29.1)	0.2167	937	26.0 (22.0–30.0)	93	26.0 (22.0–31.0)	0.3362
Alanine Aminotransferase [U/l]	10th	603	29.0 (16.0–54.5)	61	35.0 (19.0–87.0)	0.0387	944	30.5 (19.0–54.0)	92	36.5 (22.0–60.8)	0.0272
pH	50th	648	7.4 (7.3–7.4)	61	7.3 (7.3–7.4)	0.0011	959	7.4 (7.4–7.5)	93	7.4 (7.3–7.4)	0.0003
Ventilation mode	10th	631	2.0 (1.0–4.0)	61	3.0 (2.0–4.0)	0.0435	913	2.0 (1.0–4.0)	92	2.0 (1.0–4.0)	0.1234
Monocytes (%) [%]	90th	242	3.1 (1.0–7.0)	33	2.3 (1.9–4.6)	0.2979	297	3.6 (1.2–5.5)	44	2.8 (1.8–5.0)	0.3044
Pulmonary infiltrates	10th	656	101 (15%)	62	19 (31%)	0.004	961	391 (41%)	93	43 (46%)	0.3214
Sodium [mmol/l]	90th	564	141.0 (138.0–144.0)	57	141.0 (138.0–147.0)	0.109	950	142.0 (139.0–145.0)	93	143.0 (139.0–148.0)	0.015
Lactate dehydrogenase [U/l]	50th	485	356.0 (274.0–555.0)	51	601.0 (377.5–878.5)	< 0.0001	854	427.0 (322.2–563.5)	83	475.0 (368.0–720.0)	0.0012
Oxygen saturation [%]	10th	650	92.7 (90.4–94.7)	61	90.7 (87.8–92.1)	< 0.0001	959	92.0 (90.0–94.0)	93	89.0 (86.0–92.0)	< 0.0001
Red blood cell distribution width [%]	10th	654	15.0 (13.7–17.1)	62	16.0 (14.4–17.9)	0.0118	954	13.9 (13.0–15.1)	93	14.8 (13.6–16.3)	< 0.0001
Ventilation peak pressure [mbar]	50th	656	15.0 (0.0–28.0)	62	27.1 (0.0–31.0)	< 0.0001	961	0.0 (0.0–27.0)	93	0.0 (0.0–28.0)	0.0326
Height [cm]	50th	603	172.0 (165.0–180.0)	51	170.0 (165.0–178.5)	0.2775	749	175.0 (166.0–180.0)	57	175.0 (167.0–180.0)	0.392

The table shows the selected features, the number (n) of surviving and deceased patients within the next 5 days after admission having the feature available in the dataset, the median value and interquartile range (IQR) for continuous features or count (percentage) for binary features over all patients of the respective group for both non-COVID-19 and COVID-19 patients. P-values are calculated using Mann–Whitney-U non-parametric tests (continuous features) or Fisher’s exact test (binary features) between survivors and non-survivors. ^#^As data is aggregated over the first 24 h, the percentile denotes whether the 10th-, 50th- or 90th-percentile of values within the first 24 h were used.

## Discussion

We demonstrate here that lethal courses in critically ill COVID-19 patients can be predicted by a machine learning model trained on critically ill non-COVID-19 viral pneumonia patients. Furthermore, we show that the predictive performance of the model is not inferior to models developed specifically for COVID-19 patients. The plausibility of this approach is reinforced by the fact that the features that showed the highest importance in our model trained on non-COVID-19 patients and the features included in specific COVID-19 models are largely identical.

The features that are commonly included in models to predict individual mortality in COVID-19 and critically ill non-COVID-19 viral pneumonia patients can be divided in seven groups, including (1) demographic features like age and gender, (2) comorbidities like chronic obstructive pulmonary disease (COPD), obesity, hypertension and diabetes, (3) radiological signs of disease severity like multi-lobular infiltration, (4) blood infection markers and infection associated blood count parameters like C reactive protein, procalcitonin and lymphocyte counts, (5) other laboratory blood markers associated with organ distress like lactate dehydrogenase, bilirubin or blood urea nitrogen, (6) direct clinical signs of organ failure like respiratory rate, blood oxygenation or blood pressure and (7) intensive care treatment measures as indirect markers of organ failure like catecholamine doses or ventilation parameters^1–13^.

Similarly, the 20 parameters with the highest feature importance in our model trained on non-COVID-19 viral pneumonia patients included radiological signs of pulmonary infiltrates [group 3], infection-associated blood counts of neutrophils and monocytes [group 4], laboratory markers of organ distress and organ failure (thrombocytes, red blood cell distribution width, pH, P/F ratio, sodium, lactate dehydrogenase and alanine aminotransferase) [group 5], direct clinical signs of organ distress and organ failure (heart rate, blood pressure, blood oxygen saturation, urine output and respiratory rate) [group 6] or intensive care treatment measures as indirect markers of organ distress and organ failure (vasoactive inotropic score as a summary parameter of catecholamine administration, ventilation peak pressure and ventilation mode) [group 7].

While the differentiation between the latter two groups might not be sharp as the clinical signs of group 6 are always impacted by the treatment measures of group 7 and vice versa, it is clear that besides the infection parameters as the primary driving cause for mortality in viral pneumonia, all but two of the other parameters included in the 20 parameters with the highest feature importance in our model are either direct or indirect measures of organ failure and therefore represent the mechanism by which the infection induces mortality. Accordingly, the included parameters cover signs of organ distress and organ failure for all major organ systems that are in the primary focus of intensive care treatment, including heart and circulation, lungs and respiration, liver and coagulation, as well as kidneys and volume regulation.

The fact that from all demographic features [group 1] only age and height and none of the comorbidities [group 2] proved of a high enough predictive value independent of the other included parameters to show in the 20 parameters with the highest feature importance might seem unexpected at first glance, as many features from these groups have been shown in various previous studies as valuable predictors for critical and lethal courses in both critically ill COVID-19 and non-COVID viral pneumonia patients. However, when focusing on mortality, all of these features can be regarded as indirect predictors as they mediate the likelihood of specific organ failures that lead to a lethal course. Thus, in the case of the parameters included in the model that allow the prediction of lethal organ failure, the predictive value of the parameters from these first two groups of indirect parameters can be masked by the parameters indicating organ failure. For example, COPD has been shown in multiple studies to be a risk factor for a critical or lethal course in both COVID-19 and non-COVID-19 viral pneumonia patients^7,14^, but these critical and lethal courses are not caused by COPD directly and independently of organ failure. Instead, the effect of COPD is mediated through organ damage and associated increased risks of organ failure like lung or heart failure. Overall, this effect of organ failure parameters masking indirect risk factors in the prediction of lethal courses can be expected to increase with decreasing time between prediction and death. Thus, when focusing on the treatment phase in the intensive care unit, which is defined by immediate or impending organ distress and organ failure, the measures of the severity of the organ dysfunction can be expected to fully mask the indirect predictors, as we show here. The only indirect parameter that remained unmasked in our model was age, suggesting that other than the impact of specific diseases and disease groups the impact of age on organ function and compensation reserves for organ function during distress is not fully represented by the here included organ failure markers. In contrast, the role of the other parameter of the group of demographic features that was included in the 20 most important features—the patients’ height—is most probably not that the patients’ height is a predictor of mortality by itself, but that the patients’ height is an indirect prediction parameter that increases the information value of other predictors through individual normalization. As an example, the information value of urine output per kilogram of lean body weight (which is primarily determined by the height) is higher than the information value of urine output by itself.

Interestingly, the performance of the reduced model with 20 features approximated the performance of the full model towards the endpoints (Fig. 3, right), which suggests that these 20 features might indeed be closely related to the physiological processes during terminal organ failure due to viral pneumonia. However, the predictive performance of our non-COVID-19 viral pneumonia prediction models on COVID-19 patients cannot be explained by identical features alone, but it also requires similar relative weights between the features and similar value ranges at which the features exert their predictive value. This is supported by the fact that the 5 most important features from the full model on the non-COVID-19 patients training dataset (Supplementary Table 2) are also the 5 most important features of the reduced model on the COVID-19 patients test dataset and that all included 20 features show a significant feature importance on the test dataset (Fig. 4), although they were determined from the non-COVID-19 patients training data set. Interestingly, both the full and reduced models are also relatively well calibrated as shown by the relatively low Brier score (Table 2) and the calibration curve (Supplementary Fig. 1), further indicating a good transferability of the model trained on non-COVID-19 patients to COVID-19 patients. Thus, the most parsimonious explanation is that the fundamental pathophysiological mechanisms of terminal organ failure are highly similar in lethal courses of COVID-19 pneumonia and non-COVID-19 viral pneumonia^15^. Again, this is not surprising, as even if the damaging mechanisms as well as their time courses and relative rates of severe and lethal courses might differ, the signs caused by the damages can be expected to be similar. Thus, a prediction model of lethal courses for one type of viral pneumonia could be transferred to a different type of viral pneumonia and show a comparable predictive performance.

This concept of transferring knowledge obtained on one type of viral pneumonia to other types, even though they differ in mortality rates and time courses, might be highly beneficial for clinical management, especially in the context of a pandemic with a new disease. In such a situation, as experienced with COVID-19, one critical phase is the early stage of the pandemic, when the lack of knowledge and experience with the new disease puts exceptional stress on critical care resources^16^. In this situation, an effective and efficient allocation of critical care resources requires applicable prediction models of disease progression, which in the case of a new disease are only available with a significant delay. To bridge this gap and support resource allocation and disease management during the early phase of a pandemic with a new disease, it seems feasible to transfer prediction models from similar diseases for which data is widely available. As we have shown in this study, such a model transfer can lead to a similar predictive performance as models developed on early data of the specific disease.

Even though we here present only a methodological case study to demonstrate the possibility of knowledge transfer between similar diseases, models as we present here might actually be of practical use in situations when the demands on intensive care specialists overwhelm their capacities, like in the situation of a pandemic with a new disease. In such a situation, with an increasing number of patients overseen by one specialist, the risk that one patient begins to deteriorate unnoticed increases. Thus, models like the ones presented here, might allow to detect such deteriorations and to point out patients which might require a bit of focused attention while it is still early enough to intervene. Of course, our study only demonstrates that models like the one presented here hold such a potential, but further studies are required to investigate clinical processes how the allocation of intensive care specialists’ attention as a rare resource during a pandemic might be assisted by such models in a beneficial and ethical way.

This study has several limitations: since our datasets were retrospective, we could only analyze those parameters that had been recorded and documented in the electronic clinical databases. Thus, we had to exclude a variety of parameters, especially laboratory parameters like interleukins, tumor necrosis factor or specific leukocyte/lymphocyte subgroups, which might carry additional independent information or might prove as better predictors than parameters that we included, but which were not available in a sufficient number of patients of our cohorts. This also affected the number of patients that could be tested using the previously published models of Wang and Zhou, thus limiting the comparability between the here presented models.

## Conclusions

In conclusion, we have demonstrated that a machine learning model trained on critically ill non-COVID-19 viral pneumonia patients allows to predict lethal courses in critically ill COVID-19 patients within the next 5 days with a predictive performance comparable to that of models specifically developed for COVID-19 patients. Therefore, we propose for future pandemics to apply already available prediction models to support critical care resource allocation and disease management, as the transfer of knowledge seems to be feasible, even when the specific diseases differ in time courses and in rates of critical and lethal courses.

## Methods

### Ethics approval and consent to participate

Before commencement, this study was approved by the local ethics committee (Ethikausschuss 4 am Campus Benjamin Franklin, Charité—Universitätsmedizin Berlin, Chairperson Prof. R. Stahlmann, Application Number EA4/008/19, approval date: 06 Feb 2019, amendment date: 14 May 2020). All methods were carried out following relevant guidelines and regulations. As a retrospective study analyzing anonymized data from standard clinical care, without any additional data collection, informed consent was waived for this study by the competent ethics committee.

### Datasets, data extraction and data cleansing

For the non-COVID-19 dataset, we extracted all available data of patients treated between 11th November 2014 and 11th January 2021 in intensive care units (ICUs) from three centers of Charité – Universitätsmedizin Berlin, one of the largest university hospitals in Europe, with viral pneumonia and positive PCR tests for influenza, parainfluenza, metapneumovirus, orthopneumovirus or non-SARS-CoV-2 coronavirus from the computerized clinical information systems. For the COVID-19 dataset, we extracted the medical data of patients treated between 1st January 2020 and 19th April 2021 in ICUs of Charité – Universitätsmedizin Berlin with positive PCR tests for SARS-CoV-2.

The extracted medical data in both datasets comprises 123 clinical features including demographic data (e.g., age, gender, body mass index), blood gas analyses (e.g. paO2, pH, lactate), ventilation parameters (e.g. respiratory rate, tidal volume, oxygenation index), vital data (e.g., blood pressure, heart rate), laboratory tests (e.g. creatinine, bilirubin), fluid volume intake and efflux, diagnoses (ICD-10 codes), clinical scores (e.g. SOFA, SAPS2, APACHE2) and drug application rates (e.g. catecholamines).

Data cleansing included removal of invalid data (e.g. non-numeric strings instead of numbers), values outside of physical or physiological ranges and removal of duplicated values. No imputation of missing values has been performed. Patients were excluded from the data sets if their length of stay on the ICU was shorter than 24 h or if they were included in both datasets (i.e. tested positive for SARS-CoV-2 and another virus possibly causing viral pneumonia).

### Dataset preprocessing

We aimed to predict the mortality of viral pneumonia patients (formal declaration of death) within the next 5 days relative to each time point. To that end, we aggregated each patient’s time series data into time bins of 24 h, starting at the admission to the ICU. For each time bin, we calculated the median (50th percentile) and the 10th and 90th percentiles of each time-varying variable available (i.e. measured) in this time bin. Data points available before the investigated 24 h time bin were carried over and considered “current” until a new value for that variable was available. We extracted different percentiles as each of these percentiles contains different information value. For example, a short period of extreme tachycardia during the observed 24 h time window would be captured in the 90th percentile but not in the median value.

We used only features that were present in at least 30% of the time bins in the training dataset. Static variables such as age and diagnoses were then appended to each time bin, for a total of 264 features.

### Model development on non-COVID-19 patients

As we aimed to predict the mortality within the next 5 days relative to each time point, we assigned a positive class label to each of the 24 h time bins that occurred within 5 days before death of a patient. All other time bins, i.e. those that were more than 5 days before death or from surviving patients, were assigned a negative class label. To account for the different number of time bins per patient depending on how long they stayed in the ICU, we weighted each time bin by the inverse number of time bins of the respective class (positive/negative) per patient. For instance, if a patient deceased after 30 days on the ICU, we weighted the first 25 (negative) samples by 1/25 each and the last 5 (positive) samples by 1/5 each. The sample weights were used both during training and during calculation of performance metrics.

We trained gradient boosted decision tree models using XGBoost (version 1.3.3)^17^ on the training dataset of critically ill non-COVID-19 patients. To optimize the hyper-parameters of the XGBoost training algorithm, we applied a Bayesian optimization approach using the python BayesianOptimization package^18,19^ (version 1.1.0; see Supplementary Information for the parameter search range). Hyper-parameter optimization was performed using a tenfold cross-validation scheme with the average auROC value of the 10 hold-out sets (1 for each fold) as the maximization target for the optimizer (see Supplementary Table 1).

### Feature selection for reduced model

To evaluate whether a model with a reduced number of features could achieve similar performance, we performed a feature selection based on feature importances on the training dataset as measured by Shapley additive explanation (SHAP) values—a game-theoretic approach to explain the output of machine learning models using the python package SHAP (version 0.39.0)^20^. We selected the top 20 unique features with the highest average absolute SHAP values, excluding duplicates of the same physiological parameters. Using that list of features, we trained another model on the training dataset in the same way as described above for the full model.

### Prediction of mortality in COVID-19 patients and comparison with other models

To assess the ability of the gradient boosted tree models trained on non-COVID-19 viral pneumonia patients to predict the mortality of COVID-19 patients, we applied the trained model on the COVID-19 patient dataset and calculated the auROC and auPRC for the prediction of mortality, weighting each sample using the weights as described above to uniformly weigh each patient independent of their length of stay. We additionally calculated the positive predictive value (PPV)/precision, negative predictive value (NPV), F1 score, specificity and sensitivity/recall for the threshold value that yielded the highest F1 score. To assess model calibration, we determined the Brier score and calibration curves (fraction of positive vs. mean predicted value from the models). All performance metrics and the calibration curve were computed using scikit-learn (version 0.24.1). Confidence intervals were calculated using bootstrap resampling (2000 samples).

To display the time course of performance metrics, we applied the prediction models on the set of bins from the same time (e.g. first 24 h) without sample weighting, as each patient contributed at most one data point per time bin.

To compare the predictive performance of our model to those of the established clinical scores APACHE2, SAPS2 and SOFA, we calculated the respective performance metrics for the prediction of mortality within the next 5 days using the scores directly.

To further compare the predictive performance of previously published models that were specifically developed to predict mortality in COVID-19 patients^1,2,8^, we used each of the models as published (without retraining) to the COVID-19 dataset and evaluated the respective performance metrics for prediction of mortality within the next 5 days.

### Predictive performance relative to the endpoint

To determine the predictive performance of our models not only relative to the admission to the ICU but also relative to the endpoint (death in non-surviving patients), we needed to define an endpoint for the surviving patients. Using the discharge from the ICU as the endpoint would create an unrealistic and trivial comparison, as surviving patients close to discharge will show significantly different characteristics as non-surviving patients close to death. We therefore applied a case–control matching scheme: Assuming that the time courses of the diseases are comparable between the surviving and non-surviving patients, we clipped the data of each surviving patient to the length of stay of a randomly selected non-surviving patient^21,22^. Specifically, we determined the length of ICU stay for all non-surviving patients (time between admission and death). Then, for each surviving patient, we randomly picked a length of ICU stay from the non-surviving patients that was less or equal than the length of ICU stay of that surviving patient. This length of stay of the non-surviving patient then determined the endpoint for the surviving patient. We thus matched the durations of ICU treatment of the surviving patients to those of the non-surviving patients, without changing the prevalence of mortality in the dataset, as all surviving and non-surviving patients are still maintained in the dataset.

To provide a higher time resolution of the predictive performance, we aggregated data in 1 h time bins with respect to the endpoint, calculating the variable aggregates (10th, 50th and 90th percentile) as described above.

## Supplementary Information

Supplementary Information 1.

## Data Availability

No data are publicly available at this time. The trained XGBoost model is available from https://github.com/glichtner/preevent-covid19 upon request to the authors.
