# Supplementary material for: Predicting lethal courses in critically ill COVID-19 patients using a machine learning model trained on patients with non-COVID-19 viral pneumonia
Source: Sci Rep. 2021 Jun 24;11:13205. doi: 10.1038/s41598-021-92475-7 (PMC8225662; doi:10.1038/s41598-021-92475-7)
Supplement: Supplementary file 1 — Supplementary Information 1. [file 41598_2021_92475_MOESM1_ESM.pdf]

Supplementary Information

Model training parameter

Parameter group	Parameter	Range/Value	Full model value	Reduced model value
XGBoost	max_depth	2-4	3	3
	gamma	0 – 5	1.472283	0.453242
	min_child_weight	0 - 10	2.501065	2.467975
	max_delta_step	0	0	0
	subsample	0.8 – 1.0	0.971699	0.9072
	colsample_bytree	0.3 – 1.0	0.97187	0.488832
	eta	0.01 – 0.1	0.020001	0.029677
	num_boost_round	10 – 1000	979	390
	alpha	0	0	0
	lambda	1	1	1
Bayesian optimization	Initial random exploration steps	10		
	Optimization steps	100		

Supplementary Table 1: Parameters for XGBoost hyper-parameter tuning. *Shown are the hyper-parameter search ranges used to optimize the predictive performance of the prediction models using a Bayesian optimization approach and the resulting final model values.*

Model calibration

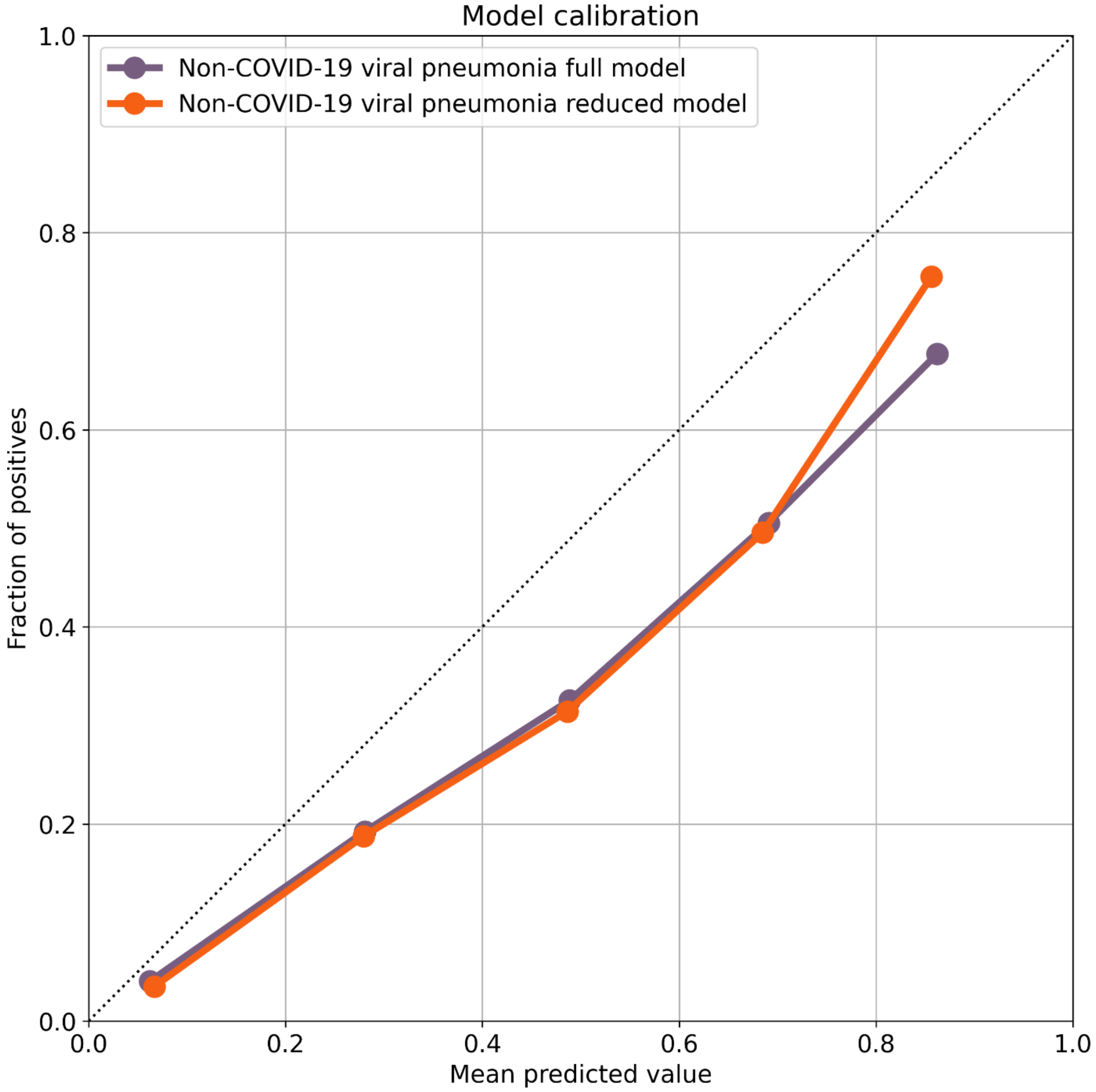

Supplementary Figure 1: Model calibration. *Shown is the observed risk of death within the next 5 days (y axis) vs the mean predicted value of the non-COVID-19 viral pneumonia model trained on all features (purple) and the non-COVID-19 viral pneumonia reduced model trained on the top 20 features (orange). Dashed line indicates perfect calibration.*

Full model feature list

FEATURE	PERCENTILE	UNIT	MEAN ABSOLUTE SHAP VALUE
**VASOACTIVE INOTROPIC SCORE**	0.5		0.21
**THROMBOCYTES**	0.1	/nl	0.21
**AGE**	0.5	a	0.19
**HEART RATE**	0.1	1/min	0.17
**NEUTROPHILS (%)**	0.1	%	0.15
**PULMONARY INFILTRATES**	0.1		0.13
**URINE OUTPUT**	0.5	ml/d	0.10
**RESPIRATORY RATE**	0.9	1/min	0.10
**PH**	0.5		0.10
**P/F RATIO**	0.1	mmHg	0.09
**VENTILATION MODE**	0.1		0.09
**MEAN ARTERIAL PRESSURE**	0.1	mmHg	0.08
**PTT**	0.1	s	0.08
**VENTILATION PEAK PRESSURE**	0.5	mbar	0.07
**RED BLOOD CELL DISTRIBUTION WIDTH**	0.9	%	0.07
**VASOACTIVE INOTROPIC SCORE**	0.9		0.07
**ALANINE AMINOTRANSFERASE**	0.1	U/l	0.07
**SODIUM**	0.9	mmol/l	0.07
**RED BLOOD CELL DISTRIBUTION WIDTH**	0.1	%	0.07
**MONOCYTES (%)**	0.9	%	0.07
**VENTILATION PEAK PRESSURE**	0.1	mbar	0.07
**PH**	0.1		0.06
**OXYGEN SATURATION**	0.1	%	0.06
**HEART RATE**	0.5	1/min	0.06
**OXYGEN PARTIAL PRESSURE (ARTERIAL)**	0.5	mmHg	0.05
**THROMBOCYTES**	0.5	/nl	0.05
**INR-QUICK**	0.9		0.05
**LACTATE DEHYDROGENASE**	0.5	U/l	0.05
**HEIGHT**	0.5	cm	0.05
**VOLUME INTAKE**	0.5	ml/h	0.05
**METABOLIC DISORDERS**	0.5		0.05
**CARCINOMA**	0.5		0.05
**EOSINOPHILS ABSOLUTE**	0.1	/nl	0.05
**ANTIMYKOTICS APPLICATION**	0.5	1/h	0.05
**NOREPINEPHRINE**	0.1	µg/kg/min	0.05
**ANTITHROMBIN III**	0.9	%	0.05
**TIDAL VOLUME**	0.1	ml	0.04
**LACTATE**	0.1	mg/dl	0.04
**LACTATE DEHYDROGENASE**	0.1	U/l	0.04
**POTASSIUM**	0.9	mmol/l	0.04
**CUMULATIVE VOLUME INTAKE**	0.5	ml/h	0.04
**CREATININE**	0.9	mg/dl	0.04

**P-RISK SCORE**	0.5		0.04
**OXYGEN PARTIAL PRESSURE (ARTERIAL)**	0.9	mmHg	0.04
**P/F RATIO**	0.5	mmHg	0.04
**THROMBOCYTES**	0.9	/nl	0.04
**HEMOGLOBIN**	0.9	g/dl	0.04
**CALCIUM**	0.1	mmol/l	0.04
**CKMB**	0.1	U/l	0.04
**P-RISK SCORE**	0.1		0.04
**VENTILATION PEEP**	0.9	mbar	0.03
**HEART RATE**	0.9	1/min	0.03
**RED BLOOD CELL DISTRIBUTION WIDTH**	0.5	%	0.03
**HYPERTENSION**	0.5		0.03
**VOLUME BALANCE**	0.5	ml/h	0.03
**INR-QUICK**	0.5		0.03
**TRIGLYCERIDE**	0.9	mg/dl	0.03
**NEUTROPHILS ABSOLUTE**	0.1	/nl	0.03
**CK**	0.5	U/l	0.03
**ALBUMIN**	0.9	g/l	0.03
**LEVOSIMENDAN**	0.1	µg/kg/min	0.03
**P/F RATIO**	0.9	mmHg	0.03
**ANTIBIOTICS APPLICATION**	0.5	1/h	0.03
**D-DIMER**	0.9	mg/l	0.03
**UREA**	0.9	mg/dl	0.03
**VENTILATION PEEP**	0.9	mbar	0.03
**C-REACTIVE PROTEIN**	0.9	mg/l	0.03
**RESPIRATORY MINUTE VOLUME**	0.1	l/min	0.03
**D-DIMER**	0.5	mg/l	0.02
**CD8 CELLS (%)**	0.1	%	0.02
**MYOGLOBIN**	0.1	µg/l	0.02
**D-DIMER**	0.1	mg/l	0.02
**NOREPINEPHRINE**	0.9	µg/kg/min	0.02
**LACTATE**	0.5	mg/dl	0.02
**PROCALCITONIN**	0.9	µg/l	0.02
**CUMULATIVE VOLUME INTAKE**	0.5	ml/d	0.02
**BILIRUBIN**	0.9	mg/dl	0.02
**C-REACTIVE PROTEIN**	0.1	mg/l	0.02
**LUNG COMPLIANCE**	0.9	ml/mbar	0.02
**BMI**	0.5	kg/m^2	0.02
**CREATININE**	0.1	mg/dl	0.02
**GLUCOSE**	0.9	mg/dl	0.02
**GLUCOSE**	0.5	mg/dl	0.02
**BICARBONATE**	0.5	mmol/l	0.02
**DIASTOLIC BLOOD PRESSURE**	0.5	mmHg	0.02
**MONOCYTES ABSOLUTE**	0.5	/nl	0.02
**ASPARTATE TRANSAMINASE**	0.1	U/l	0.02
**MONOCYTES (%)**	0.1	%	0.02

**OXYGEN PARTIAL PRESSURE (ARTERIAL)**	0.1	mmHg	0.02
**LEUKOCYTES**	0.1	/nl	0.02
**BILIRUBIN**	0.5	mg/dl	0.02
**P-RISK SCORE**	0.9		0.02
**MEAN ARTERIAL PRESSURE**	0.5	mmHg	0.02
**PTT**	0.9	s	0.02
**CKMB**	0.9	U/l	0.02
**VENTILATION PEEP**	0.5	mbar	0.02
**LACTATE**	0.9	mg/dl	0.02
**TRIGLYCERIDE**	0.1	mg/dl	0.02
**VENTILATION PEAK PRESSURE**	0.1	mbar	0.01
**TIDAL VOLUME**	0.5	ml	0.01
**NEUTROPHILS (%)**	0.5	%	0.01
**UREA**	0.1	mg/dl	0.01
**LACTATE DEHYDROGENASE**	0.9	U/l	0.01
**ASPARTATE TRANSAMINASE**	0.9	U/l	0.01
**MONOCYTES (%)**	0.5	%	0.01
**MYOGLOBIN**	0.5	µg/l	0.01
**LUNG COMPLIANCE**	0.5	ml/mbar	0.01
**CK**	0.1	U/l	0.01
**VENTILATION PEEP**	0.5	mbar	0.01
**EOSINOPHILS ABSOLUTE**	0.9	/nl	0.01
**ANTITHROMBIN III**	0.1	%	0.01
**CALCIUM**	0.5	mmol/l	0.01
**BODY TEMPERATURE**	0.9	°C	0.01
**RESPIRATORY MINUTE VOLUME**	0.9	l/min	0.01
**EOSINOPHILS (%)**	0.9	%	0.01
**CK**	0.9	U/l	0.01
**STROKE**	0.5		0.01
**VASOACTIVE INOTROPIC SCORE**	0.1		0.01
**GLUCOSE**	0.1	mg/dl	0.01
**SYSTOLIC BLOOD PRESSURE**	0.9	mmHg	0.01
**TRIGLYCERIDE**	0.5	mg/dl	0.01
**TIDAL VOLUME**	0.9	ml	0.01
**INR-QUICK**	0.1		0.01
**RESPIRATORY RATE**	0.5	1/min	0.01
**EOSINOPHILS (%)**	0.1	%	0.01
**ANTITHROMBIN III**	0.5	%	0.01
**PROCALCITONIN**	0.5	µg/l	0.01
**SODIUM**	0.1	mmol/l	0.01
**ALBUMIN**	0.1	g/l	0.01
**BASE EXCESS**	0.1	mmol/l	0.01
**EOSINOPHILS ABSOLUTE**	0.5	/nl	0.01
**VENTILATION PEEP**	0.1	mbar	0.01
**OXYGEN SATURATION**	0.5	%	0.01
**SODIUM**	0.5	mmol/l	0.01

**RESPIRATORY RATE**	0.1	1/min	0.01
**VENTILATION PEAK PRESSURE**	0.9	mbar	0.01
**NEUTROPHILS (%)**	0.9	%	0.01
**MONOCYTES ABSOLUTE**	0.1	/nl	0.01
**MONOCYTES ABSOLUTE**	0.9	/nl	0.01
**LYMPHOCYTES (%)**	0.9	%	0.01
**VENTILATION PEAK PRESSURE**	0.5	mbar	0.01
**PROCALCITONIN**	0.1	µg/l	0.01
**ALBUMIN**	0.5	g/l	0.01
**CD4 CELLS ABSOLUTE**	0.5	/nl	0.01
**EPINEPHRINE**	0.5	µg/kg/min	0.01
**PULMONARY INFILTRATES (PROGRESSIVE)**	0.9		0.01
**END TIDAL CO2**	0.5	mmHg	0.01
**FIO2**	0.1	%	0.01
**BILIRUBIN**	0.1	mg/dl	0.01
**ASPARTATE TRANSAMINASE**	0.5	U/l	0.01
**CARBON DIOXIDE PARTIAL PRESSURE**	0.1	mmHg	0.01
**END TIDAL CO2**	0.1	mmHg	0.01
**NEUTROPHILS ABSOLUTE**	0.9	/nl	0.01
**SYSTOLIC BLOOD PRESSURE**	0.5	mmHg	0.01
**TROPONIN**	0.1	ng/l	0.01
**SYSTOLIC BLOOD PRESSURE**	0.1	mmHg	0.01
**PH**	0.9		0.01
**DIURESIS**	0.1	ml/h	0.01
**LEUKOCYTES**	0.5	/nl	0.01
**WEIGHT**	0.5	kg	0.01
**VENTILATION PEAK PRESSURE**	0.9	mbar	0.01
**HEMOGLOBIN**	0.5	g/dl	0.01
**BILIRUBIN DIREKT**	0.1	mg/dl	0.01
**PULMONARY INFILTRATES (BILATERAL)**	0.9		0.01
**MYOGLOBIN**	0.9	µg/l	0.01
**PULMONARY INFILTRATES**	0.5		0.00
**DIASTOLIC BLOOD PRESSURE**	0.1	mmHg	0.00
**BPS**	0.5		0.00
**BPS**	0.9		0.00
**CARBON DIOXIDE PARTIAL PRESSURE**	0.9	mmHg	0.00
**PULMONARY INFILTRATES (BILATERAL)**	0.5		0.00
**TROPONIN**	0.5	ng/l	0.00
**CD4 CELLS (%)**	0.9	%	0.00
**BILIRUBIN DIREKT**	0.9	mg/dl	0.00
**C-REACTIVE PROTEIN**	0.5	mg/l	0.00
**BASE EXCESS**	0.9	mmol/l	0.00
**EPINEPHRINE**	0.1	µg/kg/min	0.00

**PTT**	0.5	s	0.00
**URINE OUTPUT**	0.5	ml/h	0.00
**HEMOGLOBIN**	0.1	g/dl	0.00
**LEUKOCYTES**	0.9	/nl	0.00
**DELIRIUM**	0.1		0.00
**VENTILATION PEEP**	0.1	mbar	0.00
**CHRONIC KIDNEY DISEASES**	0.5		0.00
**FERRITIN**	0.9	µg/l	0.00
**NOREPINEPHRINE**	0.5	µg/kg/min	0.00
**DIASTOLIC BLOOD PRESSURE**	0.9	mmHg	0.00
**DELIRIUM**	0.5		0.00
**BICARBONATE**	0.9	mmol/l	0.00
**BODY TEMPERATURE**	0.1	°C	0.00
**LUNG COMPLIANCE**	0.1	ml/mbar	0.00
**UREA**	0.5	mg/dl	0.00
**ALANINE AMINOTRANSFERASE**	0.5	U/l	0.00
**LYMPHOCYTES (%)**	0.1	%	0.00
**LEVOSIMENDAN**	0.5	µg/kg/min	0.00
**EOSINOPHILS (%)**	0.5	%	0.00
**BILIRUBIN DIREKT**	0.5	mg/dl	0.00
**POTASSIUM**	0.1	mmol/l	0.00
**DIURESIS**	0.9	ml/h	0.00
**CKMB**	0.5	U/l	0.00
**DELIRIUM**	0.9		0.00
**BASE EXCESS**	0.5	mmol/l	0.00
**DIURESIS**	0.5	ml/h	0.00
**OXYGEN SATURATION**	0.9	%	0.00
**POTASSIUM**	0.5	mmol/l	0.00
**ALANINE AMINOTRANSFERASE**	0.9	U/l	0.00
**CALCIUM**	0.9	mmol/l	0.00
**PULMONARY HYPERTENSION**	0.5		0.00
**CARBON DIOXIDE PARTIAL PRESSURE**	0.5	mmHg	0.00
**CUMULATIVE VOLUME BALANCE**	0.5	ml/d	0.00
**LYMPHOCYTES (%)**	0.5	%	0.00
**BODY TEMPERATURE**	0.5	°C	0.00
**FIO2**	0.5	%	0.00
**END TIDAL CO2**	0.9	mmHg	0.00
**VENTILATION MODE**	0.5		0.00
**NEUTROPHILS ABSOLUTE**	0.5	/nl	0.00
**CARDIOVASCULAR DISEASES**	0.5		0.00
**CORTICOIDS APPLICATION**	0.5	1/h	0.00
**CREATININE**	0.5	mg/dl	0.00
**BICARBONATE**	0.1	mmol/l	0.00
**LYMPHOCYTES ABSOLUTE**	0.9	/nl	0.00
**EPINEPHRINE**	0.9	µg/kg/min	0.00
**VASOPRESSIN**	0.9	E/kg/h	0.00

**DOBUTAMINE**	0.9	µg/kg/min	0.00
**ENOXIMONE**	0.9	µg/kg/min	0.00
**LEVOSIMENDAN**	0.9	µg/kg/min	0.00
**CD4 CELLS ABSOLUTE**	0.1	/nl	0.00
**CD8 CELLS (%)**	0.9	%	0.00
**ENOXIMONE**	0.1	µg/kg/min	0.00
**DOBUTAMINE**	0.1	µg/kg/min	0.00
**VASOPRESSIN**	0.1	E/kg/h	0.00
**CD8 CELLS ABSOLUTE**	0.9	/nl	0.00
**CD4 CELLS ABSOLUTE**	0.9	/nl	0.00
**PULMONARY INFILTRATES (BILATERAL)**	0.1		0.00
**PULMONARY INFILTRATES (PROGRESSIVE)**	0.1		0.00
**TROPONIN**	0.9	ng/l	0.00
**BPS**	0.1		0.00
**FERRITIN**	0.5	µg/l	0.00
**VENTILATION MODE**	0.9		0.00
**CD4 CELLS (%)**	0.1	%	0.00
**FIO2**	0.9	%	0.00
**MEAN ARTERIAL PRESSURE**	0.9	mmHg	0.00
**CD8 CELLS (%)**	0.5	%	0.00
**CD8 CELLS ABSOLUTE**	0.5	/nl	0.00
**CD4 CELLS (%)**	0.5	%	0.00
**ENOXIMONE**	0.5	µg/kg/min	0.00
**DOBUTAMINE**	0.5	µg/kg/min	0.00
**VASOPRESSIN**	0.5	E/kg/h	0.00
**SEX**	0.5		0.00
**RESPIRATORY MINUTE VOLUME**	0.5	l/min	0.00
**PULMONARY INFILTRATES (PROGRESSIVE)**	0.5		0.00
**PULMONARY FIBROSIS**	0.5		0.00
**OBESITY**	0.5		0.00
**MALNUTRITION**	0.5		0.00
**LUNG DISEASES**	0.5		0.00
**LYMPHOCYTES ABSOLUTE**	0.5	/nl	0.00
**CORONARY HEART DISEASE**	0.5		0.00
**COPD**	0.5		0.00
**PULMONARY INFILTRATES**	0.9		0.00
**ASTHMA**	0.5		0.00
**FERRITIN**	0.1	µg/l	0.00
**LYMPHOCYTES ABSOLUTE**	0.1	/nl	0.00
**CD8 CELLS ABSOLUTE**	0.1	/nl	0.00
**DIABETES**	0.5		0.00

Supplementary Table 2: Full model feature list and importances in training data set. *Shown are the features of the full model together with their importances as quantified by mean absolute SHAP values on the training dataset. For P Risk score, see* ***Supplementary Table 3****.*

P-Risk score

Condition	Score
Completely independently mobile or mostly independently mobile with no pressure ulcer present	0
Completely independently mobile or mostly independently mobile with pressure ulcer present or partially (~50%) independently mobile	1
Only marginally independently mobile or not independently mobile	2
Only marginally independently mobile or not independently mobile with continuous norepinephrine application	3

Supplementary Table 3: P-Risk Score. *The P-Risk Score is a score to measure the risk to develop pressure ulcer primarily based on an assessment of the patient's mobility. Shown are the conditions associated with the respective values of the P-Risk-Score.*
